# Cost‐effectiveness of treatments for superficial venous reflux in patients with chronic venous ulceration

**DOI:** 10.1002/bjs5.56

**Published:** 2018-05-10

**Authors:** D. Epstein, M. Gohel, F. Heatley, A. H. Davies

**Affiliations:** ^1^ Department of Applied Economics University of Granada Campus de Cartuja, 18071 Granada Spain; ^2^ Department of Vascular Surgery Addenbrooke's Hospital Cambridge UK; ^3^ Section of Vascular Surgery, Division of Surgery, Department of Surgery and Cancer, Faculty of Medicine Imperial College School of Medicine London UK

## Abstract

**Background:**

Venous leg ulcers impair quality of life significantly, with substantial costs to health services. The aim of this study was to estimate the cost‐effectiveness of interventional procedures alongside compression therapy versus compression therapy alone for the treatment of chronic venous leg ulceration.

**Methods:**

A Markov decision analytical model was developed. The main outcome measures were quality‐adjusted life‐years (QALYs) and lifetime costs per patient, from the perspective of the UK National Health Service at 2015 prices. Resource use included the initial procedures, compression therapy, primary care and outpatient consultations. The interventional procedures included superficial venous surgery, endothermal ablation and ultrasound‐guided foam sclerotherapy (UGFS). The study population was patients with a chronic venous ulcer who were eligible for either compression therapy or an interventional procedure. Data were obtained from systematic review and meta‐analysis of RCTs.

**Results:**

Surgery gained 0·112 (95 per cent c.i. −0·011 to 0·213) QALYs compared with compression therapy alone, with a difference in lifetime costs of €−1330 (−3570 to 1262). Given the expected savings in community care, the procedure would pay for itself within 4 years. There was insufficient evidence regarding endothermal ablation and UGFS to draw conclusions.

**Discussion:**

This modelling study found surgery to be more effective and less costly than compression therapy alone. Further RCT evidence is required for both endothermal ablation and UGFS.

## Introduction

Chronic venous hypertension is the most common cause of leg ulceration. The natural history is that of a continuous cycle of healing and breakdown of skin tissues over decades, causing considerable disability and impaired quality of life^1^. It has been estimated that the UK National Health Service (NHS) manages 278 000 venous leg ulcers each year, at an annual cost of €1024 million (£941 million; exchange rate £1 = €1·088^2^), mostly in primary care and community nursing services^3^. Moreover, with an ageing and increasingly overweight population, the prevalence of venous ulceration is likely to increase.

Compression with multilayer bandaging is standard therapy, with the aim of improving venous return and reducing venous hypertension until the ulcer has healed, followed by graduated compression stockings for life to prevent recurrence^1^ ^4^. However, compression stockings are uncomfortable and patient compliance is poor. Many non‐surgical therapies have been proposed as alternatives or complements to compression to promote faster healing (manuka honey, larval therapy, antibiotics, infrared light, ultrasound and many more), but with limited effectiveness^1^. Cost‐effectiveness analysis can aid decision‐makers to provide therapies that offer good value for money. Although many cost‐effectiveness studies of non‐surgical therapies have been published^5^ so far, no cost‐effectiveness analyses of surgical procedures *versus* compression therapy have been conducted. Recent reviews^6^ ^7^ concluded that superficial venous surgery alongside compression therapy did not promote faster healing but did reduce recurrence. Surgery requires a large upfront cost, but the benefits in terms of fewer recurrences may take some years to materialize. Decision models provide a framework for comparing the risks and rewards of different options over an appropriate time horizon. As well as superficial venous surgery, several other interventional treatment options are available, such as endothermal treatments (endovenous laser ablation (EVLA) and radiofrequency ablation (RFA)), and ultrasound‐guided foam sclerotherapy (UGFS). These have shown excellent results in the treatment of chronic venous disease without ulceration^1^ ^8^, ^9^. However, there is very little RCT evidence about their effectiveness in treating venous leg ulcers. One RCT^10^ has been conducted for EVLA, which was found to be more effective than compression for both healing and preventing recurrence. This trial was included in the systematic review by Mauck and colleagues^7^, but was considered to be of insufficient methodological quality to be included in the Cochrane review^11^. The review by Mauck *et al*.^7^ found one RCT for UGFS, and concluded it was no better than compression for healing; the trial^12^ did not report recurrence.

The aim of this study was to estimate the cost‐effectiveness of interventional procedures alongside compression therapy *versus* compression therapy alone for treatment of chronic venous leg ulcers using a decision model. The primary (base case) study compared superficial venous surgery with compression. Scenario analyses incorporated the limited evidence from other interventional procedures, including EVLA^10^ and UGFS^12^.

## Methods

### Decision model

#### *Model overview*

Analyses were performed from the perspective of the UK NHS and Personal Social Services at 2015–2016 prices. Currency conversion was made using purchasing power parities, at a rate of £1 = €1·088^2^. Discounting at 3·5 per cent per year was applied for costs and QALYs (varied in sensitivity analysis)^13^. The study was reported according to guidelines for economic evaluation^14^.

A Markov decision model was used to carry out the analyses. A decision model is a mathematical tool that links together the different types of evidence that might be of interest to decision‐makers in a coherent structure. In the context of venous leg ulcers, the relevant outcomes include the effectiveness of the treatments (in terms of healing and recurrence), the costs over the short and long term, and the impact of the treatments and the disease on health‐related quality of life (HRQoL). Clinical trials form the evidence base for such models, yet RCTs usually have follow‐up limited to a few years. As venous leg ulceration is a chronic condition, and can recur several times, a long decision horizon is appropriate. Decision models provide a framework for extrapolating outcomes beyond the trial reporting period.

#### *Population*

The population of this study was patients with a chronic venous leg ulcer who would be eligible for an interventional procedure or compression therapy alone. A chronic venous leg ulcer is defined as an open lesion between knee and ankle joint that has remained unhealed for at least 6 weeks and occurs in the presence of venous disease^1^ ^9^. Patients are usually elderly. In the ESCHAR study^15^, for example, the mean age was 73 (i.q.r. 60–80) years, and around 60 per cent of the patients were women.

#### *Interventions and comparators*

The comparator was compression therapy alone. There are many variations of compression therapy^16^, but in this study it was assumed that multilayer bandaging aiming to provide 40 mmHg of compression at the ankle was used until the ulcer had healed, followed by the use of compression stockings for life to prevent recurrence. In the interventional procedure arm, compression therapy was applied as in the comparator arm, but, in addition, surgery was used to treat the superficial venous reflux as soon as possible.

#### *Health states*

**Figure 1:**
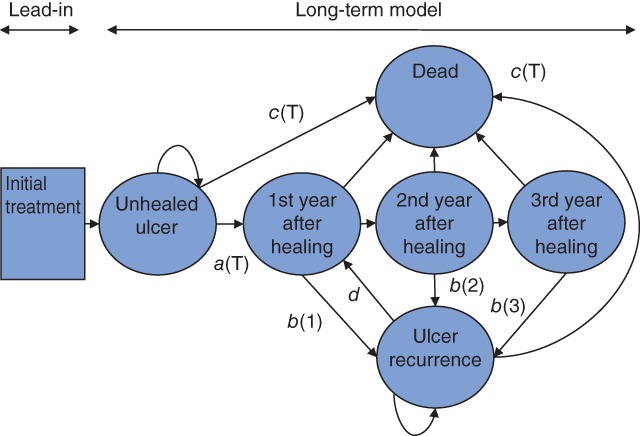
Markov model structure. The lead‐in period is 6 weeks, during which initial therapy is undertaken. Subsequent cycles in the long‐term Markov model are 1 year. Transitions: a(T) is the rate of healing at time T; b(1), b(2) etc. are the rates of recurrence in the first, second, etc. year after healing; c(T) is the mortality rate at time T; d is the rate of healing after recurrence. Tunnel states for the fourth year and beyond after healing are included in the model but not shown in the figure. See Appendix S4 (supporting information) for a full description of the model states and transitions

The two main effectiveness outcomes captured in the model are ulcer healing and recurrence. *Fig*. 1 shows the model structure in the form of an influence diagram. Full details of the model structure are described in *Appendix* S1 (supporting information). There is a lead‐in period representing the time from initiating therapy to first follow‐up (6 weeks), during which compression bandaging is applied, diagnosis of venous ulcer is confirmed, and initial surgery is undertaken, depending on the protocol of that treatment arm. This period includes recovery time from surgery. Patients then enter a ‘long‐term’ state transition Markov model^17^ with a cycle length of 1 year. The model allows ulcers to heal and recur, perhaps several times over the lifetime. The starting age was 73 years, and the model estimated outcomes up to 100 years of age. Rates of healing and rates of recurrence can vary over time. Mortality rates increase with age, but are assumed not to differ between treatments or between states. Rates are obtained from the literature and are converted to annual transition probabilities using a published method^18^ (*Appendix* S2, supporting information). The model was constructed in Excel^®^ (Microsoft, Redmond, Washington, USA). A copy is made available to researchers under a CC BY 4.0 licence (https://doi.org/10.17632/7634sv27zp.1).

### Parameter estimates for the model

#### *Relative treatment effects for healing and recurrence*

A clinical literature review^7^ estimated the relative treatment effects for healing for surgical procedures *versus* compression therapy. The systematic review included both observational studies and RCTs, but in the present study only results obtained from RCT data were included in the model, as the observational studies were assessed as of low methodological quality with a high risk of bias^7^. The results of the meta‐analysis of RCT data are shown in *Table* 1. Superficial venous surgery did not promote faster healing than compression bandaging alone (risk ratio (RR) 1·04, 95 per cent c.i. 0·98 to 1·09). The clinical review^7^ found that superficial venous surgery led to fewer recurrences than compression alone, although the overall (pooled) result did not reach statistical significance (RR 0·67, 0·41 to 1·10).

**Table 1:** Risk ratios estimated by a published systematic review^7^

	Mean RR
Ulcer healing (RR > 1 favours intervention)	
Superficial venous surgery + compression *versus* compression alone (5 RCTs)	1·04 (0·98, 1·09)
EVLA + compression *versus* compression alone (1 RCT)	3·40 (1·65, 6·98)
UGFS + compression *versus* compression alone (1 RCT)	0·86 (0·58, 1·28)
Recurrence (RR < 1 favours intervention)	
Superficial venous surgery + compression *versus* compression alone (2 RCTs)	0·67 (0·41, 1·10)
EVLA + compression *versus* compression alone (1 RCT)	0·03 (0·00, 0·58)

Values in parentheses are 95 per cent confidence intervals. RR, risk ratio; EVLA, endovenous laser ablation; UGFS, ultrasound‐guided foam sclerotherapy.

The evidence on EVLA was limited to a single RCT^11^, which found substantially faster healing with EVLA than for compression (RR 3·40, 95 per cent c.i. 1·65 to 6·98). Furthermore, in the 22 patients with a healed ulcer following EVLA, there were no cases of recurrence of ulceration, whereas with compression therapy alone there were four recurrences in nine healed patients (RR 0·03, 0·00 to 0·58)^10^. However, the Cochrane group^11^ excluded this study for poor methodological quality, and thus it was not included in the base case model.

UGFS did not show more rapid healing than compression (RR 0·86, 95 per cent c.i. 0·58 to 1·28), although the trial^12^ was unable to recruit the required number of patients and so these results should be interpreted with caution. The RCT followed up patients only to 24 weeks, which was inadequate to assess recurrence. Although case series^19^ ^20^ have suggested recurrence rates might be similar to those following surgery, these studies presented a high risk of bias and are therefore not sufficient evidence for the model. Given the lack of strong evidence of any benefit, it was assumed that recurrence rates after UGFS are the same as for compression.

#### *Rate of healing with compression therapy alone*

**Figure 2:**
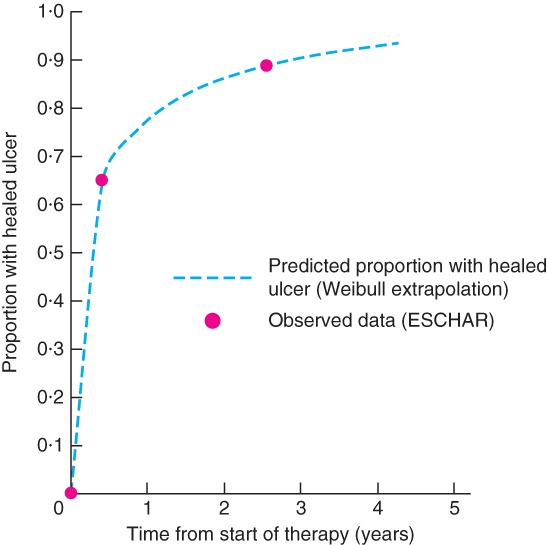
Proportion of patients with healed ulcer observed in the ESCHAR trial^21^ (compression therapy alone arm): observed data and proportion predicted using the Weibull function

The ESCHAR trial^21^ was a large, UK‐based, publicly funded RCT with a long follow‐up; these results are used to inform the natural history of venous leg ulcers with compression therapy (the comparator in the model). *Fig*. 2 shows the observed proportion of patients with a healed ulcer at 6 months (66 per cent) and 3 years (89 per cent) in the compression therapy‐alone arm of the trial^21^. A constant rate (exponential survival) model is inappropriate for these data. The rate of healing slowed over time: most ulcers healed within the first 6 months with compression therapy, but a small proportion of patients had very long healing times. This time‐dependent pattern of healing can be modelled with a Weibull distribution (*Table* 2; *Appendix* S2, supporting information). The clinical trial did not report any measure of statistical uncertainty (such as standard error) associated with the proportion healed, so the corresponding standard error of the Weibull parameters cannot be estimated. However, given the large sample size of ESCHAR (257 patients randomized to compression alone and only 27 lost to follow‐up), the statistical error was likely to be very small. It was assumed for the probabilistic sensitivity analysis that the standard errors of the Weibull parameters were 10 per cent of the means.

**Table 2:** Rates of healing and recurrence with compression therapy used in the model, based on ESCHAR^21^

Event rate in the model	Data from ESCHAR (compression therapy arm)
Rate of ulcer healing with compression therapy	65% healed after 6 months and 89% healed after 3 years
Rate of recurrence with compression therapy	28% recurred after 1 year and 56% recurred after 4 years
Rate of healing after recurrence	89% healed 3 years after onset of recurrence

#### *Rate of recurrence with compression therapy alone*

**Figure 3:**
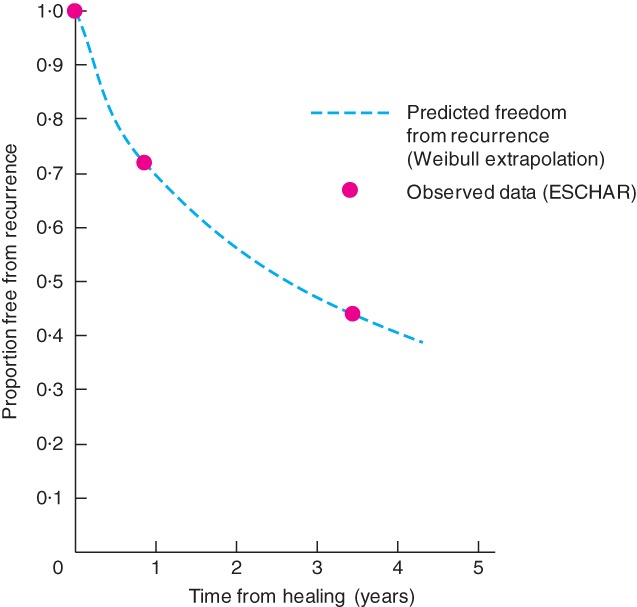
Freedom from recurrence observed in the ESCHAR trial^21^ (compression therapy alone arm): observed data and proportion predicted using the Weibull function

The ESCHAR trial^21^ found the probability of recurrence after compression therapy was 28 per cent after 1 year and 56 per cent after 4 years (*Fig*. 3). The rate of recurrence of venous ulceration slowed over time: recurrence was more likely in the first year than in the second year after healing, and so on. Therefore, the rate of recurrence used in the model was also estimated from these data using a Weibull distribution. The ESCHAR data were not reported exactly in the format required by the model. The model requires the time from healing to recurrence, whereas the clinical trial reported time from randomization to recurrence (*Appendix* S1, supporting information). However, in practice, the difference is minor. One‐third of ulcers in the ESCHAR trial were already healed at baseline, and two‐thirds of the remainder had healed by 6 months. Hence it is assumed that the ESCHAR data were approximately indicative of the recurrence rate from time of healing.

#### *Mortality*

The ESCHAR trial^21^ found that 17 per cent of patients had died by 3 years, with no statistically significant difference between groups, or any mortality associated with surgery. This rate of mortality was greater than would be expected in the general population of the same age, reflecting the greater co‐morbidity associated with venous disease. Mortality rates in the model were based on life‐table estimates from a general population of the same age^22^, but calibrated upwards to coincide with the average mortality observed in ESCHAR.

#### *Resource use and unit costs*

NHS resources included two bandage changes per week (40‐min community nurse home visit^23^ ^24^ and wound care consumables^25^), general practitioner and primary care nurse consultations associated with the ulcer^23^ ^24^, ^26^, hospital outpatient visits^24^ ^26^ and interventional procedures^27^ (*Table* 3). Compression stockings are used to prevent recurrence after the ulcer has healed^25^. It was assumed there were no other ulcer‐related healthcare expenses once the ulcer had healed. Costs of surgery, UGFS and EVLA procedures were obtained from the CLASS (Comparison of LAser, Surgery and foam Sclerotherapy) study^27^. The prices of the catheter, laser fibre and other kit for EVLA in the CLASS study were estimated from prices paid by the lead centre, €278; these are usually negotiated with the supplier.

**Table 3:** Resource use and unit costs used in the model

	No. of patients in sample	Use per patient*	Unit cost	Total cost per patient*	References
Compression therapy related					
Bandage (Coban™ 2) + dressing (UrgoTul^®^)		Applied by community nurse twice‐weekly until healing (40‐min home visit)	€8·80 (bandages) + €1·63 (dressing) + nurse visit €73 per h	€118 per week	23–26
Compression stockings		Applied after healing, changed every 3 months	€34·02	€34 every 3 months	25
Other healthcare related to ulcer while healing†					
GP consultation	169	2·32(7·60)	€44 per visit	€103 per year	23, 26
Nurse consultation in GP surgery (22 min)	169	25·32(35·29)	€61 per h	€566 per year	23, 26
Hospital outpatient visits‡	169	8·84(19·01)	€77 per visit	€683 per year	23, 26
Cost of interventional procedures					
Surgery	195			€997(448)	27
EVLA	183			€802(222)	27
UGFS	182			€267(175)	27

* Values are mean or mean(s.d.);

† number of visits per year;

‡ without ultrasound imaging. Coban™ 2 (3M, St Paul, Minnesota, USA); UrgoTul^®^ 5 × 5 cm (Urgo Medical, Chenôve, France). GP, general practitioner; EVLA, endovenous laser ablation; UGFS, ultrasound‐guided foam sclerotherapy.

#### *Health‐related quality of life*

Clegg and Guest^28^ estimated the mean HRQoL (utility) associated with an unhealed venous leg ulcer to be 0·64 (95 per cent c.i. 0·60 to 0·68) by standard gamble from 200 members of the general public (some of whom had personal experience of ulcers). They assumed that, once the ulcer had healed, patients returned to full health for a person of that age.

Carradice and co‐workers^29^ reported HRQoL and time to return to normal activities after varicose vein surgery and EVLA. Time to return to work or normal activity was longer after surgery than EVLA (14 *versus* 4 days respectively; *P* < 0·001) and both procedures resulted in reduced HRQoL at 1 week compared with baseline (reduction in HRQoL measured by the EQ‐5D™ (EuroQol Group, Rotterdam, The Netherlands) index of 0·05; *P* = 0·024). In the model, it was assumed that the reduction in HRQoL was 0·05, lasting for 2 weeks after surgery and for 4 days after EVLA.

### Cost‐effectiveness analysis

The results of the analyses were presented as estimates of mean total cost per patient and mean QALY for each therapy option. The following univariable sensitivity analyses were conducted to test the robustness of the results to alternative input data: no difference between surgery and compression in time to healing; no difference between surgery and compression in time to recurrence; an unhealed ulcer causes much higher detriment to HRQoL (utility 0·5); an unhealed ulcer causes little detriment to HRQoL (utility 0·9); only one bandage change per week; discount rate of 0 per cent and discount rate of 6 per cent per year.

Probabilistic sensitivity analyses were conducted using 1000 Monte Carlo simulations^30^. *Appendix* S3 (supporting information) shows the distributions of model parameters used in the probabilistic sensitivity analysis. Analysis of co‐variance was used to identify the input parameters that most explained the overall variance in difference in costs and QALYs predicted by the model^30^. Exploratory analyses were also conducted to evaluate the cost‐effectiveness of EVLA and UGFS, using the effectiveness estimates from Viarengo *et al*.^10^ and O'Hare and Earnshaw^12^.

## Results

### Base case analysis

**Figure 4:**
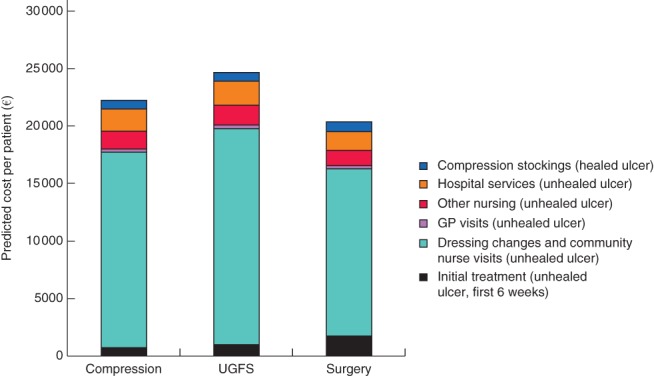
Predicted total mean cost per patient over the lifetime (undiscounted, euros). Compression stockings are used to prevent recurrence and are assumed to be changed every 3 months. Hospital services include admissions and outpatient visits related to the unhealed leg ulcer. General practitioner (GP) and other nursing are visits to primary care related to the unhealed leg ulcer. Dressings and bandages are assumed to be changed twice‐weekly by a district nurse until the wound is healed. The initial treatment is surgery, ultrasound‐guided foam sclerotherapy (UGFS) or compression only

**Figure 5:**
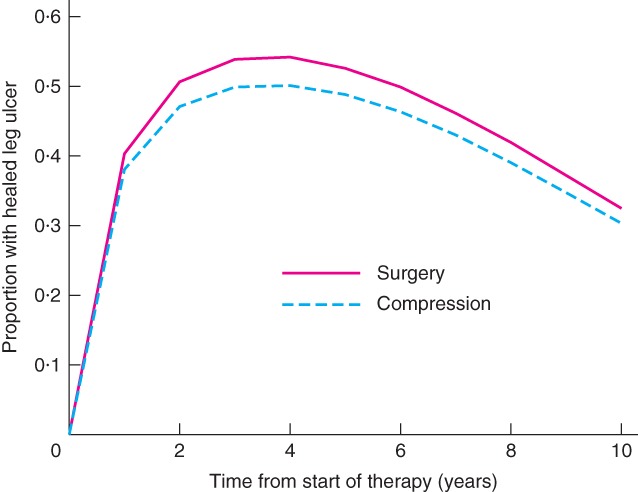
Estimated proportion of patients with a healed leg ulcer following surgery or compression therapy alone

The results of the cost‐effectiveness model are shown in *Table* 4. Surgery was more effective and less costly over the lifetime of the patient. Surgery gained 0·112 (95 per cent c.i. −0·011 to 0·213) QALYs compared with compression therapy alone, with a difference in lifetime costs of €−1330 (−3570 to 1262). The (undiscounted) cost of compression therapy over the lifetime of a patient with a venous leg ulcer was more than €22 000, of which over 75 per cent was the cost of bandaging and nursing while the ulcer healed. Surgery reduced considerably the probability of recurrence, and hence was cost‐saving overall (*Fig*. 4). The savings in community care would begin to outweigh the initial cost of the surgical procedure after 4 years (*Fig*. S1, supporting information). *Fig*. 5 shows the proportion of patients predicted by the model to have a healed ulcer at each year after start of therapy.

**Table 4:** Results of base case analysis of surgery versus compression only and exploratory analyses with endovenous laser ablation and ultrasound‐guided foam sclerotherapy

	Discounted total QALY per patient		Discounted total lifetime cost per patient (€)	
	Mean	Mean difference from compression	Mean	Mean difference from compression
Base case analysis				
Compression only	5·878	0·000 (reference)	19 046	0·000 (reference)
Surgery	5·990	0·112 (−0·011, 0·213)	17 717	−1330 (−3570, 1262)
Exploratory analyses of the cost‐effectiveness of other interventions*				
UGFS	5·789	−0·089 (−0·364, 0·121)	21 104	2057 (− 2197, 7660)
EVLA	6·653	0·775 (0·476, 1·033)	4027	−15 020 (−20 620, −9171)

Values in parentheses are 95 per cent confidence intervals.

* Based on results of RCTs^10^ ^12^ of low methodological quality. QALY, quality‐adjusted life‐year; UGFS, ultrasound‐guided foam sclerotherapy; EVLA, endovenous laser ablation.

### Univariable and probabilistic sensitivity analysis, and analysis of co‐variance

Univariable sensitivity analyses alter one input to the model, leaving the others the same as the base case (*Table* 5). In one sensitivity analysis, assuming there was no difference in recurrence rates between treatments, surgery was both more effective and more expensive than compression bandaging, and the incremental cost‐effectiveness ratio was €27 647 per QALY. Surgery was still more effective in the model than compression alone, because the base case RR for healing was slightly in favour of surgery, although the effect was not statistically significant (RR 1·04, 95 per cent c.i. 0·98 to 1·09). Evidently, if surgery had no positive effect on either healing or recurrence, compression would be the cheaper and more effective option.

**Table 5:** Results of univariable sensitivity analyses

	Sensitivity analyses	Most effective option	Option with lowest cost	ICER (surgery *versus* compression)
Base case		Surgery	Surgery	Surgery dominant
RR for healing after surgery *versus* compression is 1·04 (0·98, 1·09)	RR for healing: 1	Surgery	Surgery	Surgery dominant
RR for recurrence after surgery *versus* compression is 0·67 (0·41, 1·10)	RR for recurrence: 1	Surgery	Compression	€27 647 per QALY
EQ‐5D™ score associated with unhealed ulcer is 0·64 (0·60, 0·68)	EQ‐5D™ score: 0·5	Surgery	Surgery	Surgery dominant
EQ‐5D™ score associated with unhealed ulcer is 0·64 (0·60, 0·68)	EQ‐5D™ score: 0·9	Surgery	Surgery	Surgery dominant
Two bandage changes per week until ulcer healed	1 bandage change per week until ulcer healed	Surgery	Surgery	Surgery dominant
Discount rate 3·5%	0% or 6%	Surgery	Surgery	Surgery dominant

Values in parentheses are 95 per cent confidence intervals. ICER, incremental cost‐effectiveness ratio; RR, risk ratio; QALY, quality‐adjusted life‐year.

The probabilistic sensitivity analysis found that, using the base case inputs to the model, surgery had a probability in excess of 0·90 of being the most cost‐effective option at any cost‐effectiveness threshold. Analysis of co‐variance indicated that the RR for recurrence is the input parameter that explains most (over 86 per cent) of the overall uncertainty in incremental cost and incremental QALYs between surgery and compression therapy alone (*Table* S1, supporting information).

### Exploratory analysis of EVLA and UGFS for treating venous leg ulcers

Exploratory analysis indicated that, if the RCT estimates of healing and recurrence rates from Viarengo *et al*.^10^ and O'Hare and Earnshaw^12^ were accurate, EVLA would be both more cost‐saving and more effective than surgery, and UGFS would be more costly and less effective (*Table* 4).

### Validation of the model

It is important to validate a model by comparing predictions against observed data. The model predicted the death of 17 per cent of patients (the same in each treatment group), and the mean ulcer‐free time was 89 weeks in the surgery group *versus* 83 weeks with compression therapy alone at 3 years (after half‐cycle correction). The ESCHAR study found that 16 per cent of patients in the compression‐only group died and 19 per cent in the surgery group (*P* = 0·245), and the ulcer‐free time at 3 years was 100 weeks with surgery and 85 weeks with compression alone. The model predictions are not exactly the same as in the ESCHAR trial, because the model incorporates clinical risk evidence from diverse sources (the ESCHAR trial and meta‐analysis of RRs).

## Discussion

This study estimated the cost‐effectiveness of compression therapy alone *versus* interventional procedures (alongside compression therapy) for the treatment of venous leg ulcers. The main finding was that surgery is the most effective and least costly treatment option. Venous leg ulcers are very costly for health services to treat and, by preventing recurrence, surgery would pay for itself within 4 years, compared with compression therapy alone. The results are robust to alternative assumptions. If the outcomes of the study by Viarengo and colleagues^10^ were confirmed, EVLA would be very cost‐effective. However, given the risk of bias in that RCT, no definitive conclusions can yet be reached.

This is the first cost‐effectiveness analysis to compare surgical procedures with compression therapy for venous leg ulcers. The data for RRs were based on a systematic review of RCTs^7^, which are usually considered to be the most valid form of evidence. At the time of publication of the systematic review^7^, the ESCHAR RCT^21^ reported substantially fewer recurrences after surgery at 4 years, whereas van Gent and co‐workers^31^ showed no difference in recurrence at 2 years. However, recently published long‐term follow‐up from the latter study showed that surgery almost halved the probability of recurrence at 10 years^32^, corroborating the outcome of the ESCHAR trial^21^. Nevertheless, these data should be interpreted cautiously. The systematic reviewers rated the RCTs as having a moderate risk of bias, mainly due to omissions in the reporting of the method of randomization, blinding, how missing data were handled, and the funding source.

In some of the older RCTs, such as ESCHAR, many patients in the interventional arm either did not have surgery or underwent procedures that would be considered suboptimal by modern endovenous standards^33^. The model assumes patients are maintained on lifetime compression therapy after interventional procedures to avoid recurrence. It has been suggested^33^ that some patients could be managed successfully without compression after interventional procedures. However, these considerations would make interventional procedures even more cost‐effective than compression alone.

One common treatment modality not included in this study is endovenous RFA. This was owing to a lack of published randomized trials using RFA in patients with chronic venous ulcers. However, the mode of action and published technical success rates are comparable to those for EVLA^34^ ^35^, leading some bodies (including the UK National Institute for Health and Care Excellence) to describe EVLA and RFA together as endovenous thermal ablation procedures^36^.

No other cost‐effectiveness analyses have compared interventional procedures with compression therapy. VenUS I estimated the annual cost of compression therapy with multilayer bandaging to be €1412 (95 per cent c.i. 1291 to 1600) (£1298, 1187 to 1471) at 2001 prices^16^. The present study suggests the first‐year cost of compression therapy is closer to €4200 at 2015–2016 prices. Inflation accounts for one‐quarter of the difference^37^, and the remainder may be due to the assumption of two bandage changes per week, with no washing and reuse of bandages. Sensitivity analysis using one bandage change per week, rather than two, did not change the main findings.

Further research would be worthwhile in several areas. First, the analysis indicated that the RR for recurrence is the most influential parameter in this decision. There may be different impacts by subgroups. ESCHAR found that the greatest relative benefit of surgery tended to be in patients with isolated superficial venous reflux. Further RCTs, or meta‐analysis of existing RCTs using individual patient data, might investigate whether differing patterns of venous reflux or other factors influence outcomes. Second, further RCTs should compare interventional procedures with one another as well as, or instead of, compression therapy. Endothermal procedures might be extremely effective for the treatment of venous leg ulcers, but this evidence is still weak. There is currently an ongoing RCT comparing early endovenous ablation (EVLA, RFA or UGFS) with delayed endovenous ablation for the treatment of venous leg ulcers (Early Venous Reflux Ablation (EVRA) trial; ISRCTN02335796). Once finalized, these results will inform the optimal use and timing of endovenous procedures in the management of leg ulcers.

The results of this study should inform the next generation of clinical guidelines for venous leg ulcers. In a very challenging economic climate, the delivery of surgical procedures to patients with chronic venous ulcers would require significant changes to current pathways of care. These patients usually present to and are managed by community nursing teams in the very peripheries of healthcare systems, whereas surgery, specialist venous scanning and endovenous interventions are usually delivered in secondary care environments. Published guidelines should be supported by plans for implementation and robust audit frameworks.

## Supporting information


**Appendix S1** Model structure
**Appendix S2** Weibull model for estimating rates of events
**Appendix S3** Probabilistic sensitivity analysis
**Appendix S4** Calculating probabilities from rates
**Table A1** The transition rate matrix Q
**Table A2** The transition rate matrix Q, simplified to exclude tunnel states
**Table A3** Eigenvalues matrix D
**Table A4** Representation of matrix D using intermediate variables
**Table A5** Exponential of D
**Table A6** Eigenvectors matrix U
**Table A7** Representation of matrix U using intermediate variables
**Table A8** Inverse of U = U^‐1^
**Table A9** Probability matrix P
**Table A10** Probability matrix P, expressed in terms of intermediate variables
**Table A11** Reconstituted 8x8 probability matrix with tunnel states
**Fig. S1** Incremental cost‐effectiveness ratio (ICER; cost per quality‐adjusted life‐year gained) over time. Difference in cost: difference in overall cost in euros per patient between surgery and compression therapy only. Dominates: the overall costs of surgery are lower and the health gain is greater than compression therapy alone
**Table S1** Results of analysis of co‐variance
