# Supplementary material for: Cost‐effectiveness of treatments for superficial venous reflux in patients with chronic venous ulceration
Source: BJS Open. 2018 May 10;2(4):203–12. doi: 10.1002/bjs5.56 (PMC6069357; doi:10.1002/bjs5.56)
Supplement: Supplementary file 1 — Appendix S1 Model structure Appendix S2 Weibull model for estimating rates of events Appendix S3 Probabilistic sensitivity analysis Appendix S4 Calculating probabilities from rates Table A1 The transition rate matrix Q Table A2 The transition rate matrix Q, simplified to exclude tunnel states Table A3 Eigenvalues matrix D Table A4 Representation of matrix D using intermediate variables Table A5 Exponential of D Table A6 Eigenvectors matrix U Table A7 Representation of matrix U using intermediate variables Table A8 Inverse of U = U‐1 Table A9 Probability matrix P Table A10 Probability matrix P, expressed in terms of intermediate variables Table A11 Reconstituted 8x8 probability matrix with tunnel states Fig. S1 Incremental cost‐effectiveness ratio (ICER; cost per quality‐adjusted life‐year gained) over time. Difference in cost: difference in overall cost in euros per patient between surgery and compression therapy only. Dominates: the overall costs of surgery are lower and the health gain is greater than compression therapy alone Table S1 Results of analysis of co‐variance [file BJS5-2-203-s001.docx]

**BJS5_56**

**Cost-effectiveness of treatments for superficial venous reflux in patients with chronic venous ulceration**

**D. Epstein, M. Gohel, F. Heatley and A. H. Davies**

**Appendix S1** Model structure

The two main effectiveness outcomes captured in the model are ulcer healing and recurrence. *Fig. 1* (in the main paper) shows the model structure in the form of an influence diagram. There is a lead-in period representing the time from initiating therapy to the first follow-up (6 weeks), during which compression bandaging is applied and initial surgery undertaken, depending on the protocol of that treatment arm. This period includes recovery time from surgery. Patients then enter a “long-term” state-transition Markov model[1]. The cycle length represents discrete time periods during which rates of events are assumed to be constant. In this model, the cycle length is one year. An unhealed ulcer may heal during any of the Markov model cycles, with rate *a*(*T*). The notation *T* indicates that the rate can change with time from the start of the model, or with age.

This model allows ulcers to heal and recur several times over the lifetime. Once healed, the ulcer may recur during any cycle with rate *b*(*t*). The rate of recurrence can change over time, but time *t* in this case does not refer to calendar time (*T*), but rather the time from healing, which will vary between patients. To achieve this semi-Markov property, the model incorporates a series of tunnel states (1^st^ year after healing, 2^nd^ year etc., up to 5^th^ year after healing). Consequently, *b*(1) refers to the rate of recurrence during the coming year in a leg whose ulcer healed up to one year ago, *b*(2) refers to the rate of recurrence during the coming year in a leg whose ulcer healed between one and two years ago, and so on. It is assumed that recurrence rates are constant in patients whose ulcer healed more than 5 years ago. Rates a(T) and b(t) vary depending on the initial treatment.

A recurrent ulcer will heal at rate *d* (*Fig. 1*). This rate of healing is assumed not to depend on the initial treatment and not to vary with time. Relaxing these two assumptions would considerably complicate the state-transition model. Patients may die during any cycle, with rate *c*(*T*). Mortality rates increase with age, but are assumed not to differ between treatments or between states. Rates are obtained from the literature (*Appendix S2*) and are converted to annual transition probabilities using a published method (*Appendix S4*) [2]. The model is constructed in Excel.

**Appendix S2** Weibull model for estimating rates of events

A Weibull model has two parameters λ and ρ, and the proportion healed S(T) at time T is given by the formula S(T)= exp(-λTρ). Given the Kaplan-Meier estimate from ESCHAR of the proportion healed S(T) at follow-up T=0.5 years and T=3 years, we estimated these two parameters by forming two equations of the general form log[S(T)] = -λTρ and solving them simultaneously (see Table below).

Given the parameters λ and ρ, the rate of healing q(T) at time T with compression therapy is given by the Weibull formula q(T, compression)= λρ(T)^(ρ-1)^.

The systematic review[3] reported risk ratios of surgery versus compression therapy alone. The rate of healing at time T after surgery, assuming proportional hazards, is q(T, surgery)= rλρ(T) ^(ρ-1)^, where r is the risk ratio obtained from the metanalysis.

| Event rate in the model | Data reported by ESCHAR (probability of event) | Parameters used in the model, estimated from the ESCHAR data |
| --- | --- | --- |
| Rate of ulcer healing with compression therapy, events/person-year | 65% healed after 6 months and 89% healed after 3 years | Weibull rate of healing, λ= 1.4 and ρ = 0.415 |
| Rate of recurrence with compression therapy, events/person-year after healing | 28% recurred after 1 year and 56% recurred after 4 years | Weibull rate of recurrence, λ = 0.329 and ρ = 0.661 |
| Rate of healing after recurrence, events/person-year | 89% healed after 3 years | Healing rate assumed constant, λ=0.736 / person-year |

**Appendix S3** Probabilistic sensitivity analysis

Probabilistic sensitivity analyses were conducted using 1000 Monte-Carlo simulations[4]. Resource use and cost data were assigned gamma distributions with the mean and SE in *Table 3* in the main paper[4]. The utility associated with an unhealed ulcer (expressed as decrement from full health) was assigned a gamma distribution with the mean and 95% CI given in the main text. For a variable assumed to be distributed gamma(α,β), with data available about the sample mean Φ and standard error s, the parameters were estimated α= Φ^2^/s^2^, and β=s^2^/Φ[4].

Risk ratios of surgical therapies, compared with compression therapy alone, were assigned lognormal distributions with the mean and 95% CI given in *Table 1* in the main paper. For a variable assumed to be distributed lognormal(μ,σ^2^), with data available about the sample mean Φ and a 95% confidence interval (*lower*, *upper*) for Φ, the parameters of the distribution were estimated by σ^2^=[log(*upper*)– log(*lower*)]/(1.96x2), and μ= log(Φ) [4].

**Appendix S4** Calculating probabilities from rates

This appendix describes the procedure used to calculate annual probabilities required by the Markov model from the rates of events compiled in the literature review. The procedure is shown as a series of five steps, corresponding to the algorithm outlined in Jones, Epstein and Garcia-Mochon, 2017[2].

A rate is an instantaneous quantity expressed as the number of events per patient-year. *Table A1* shows the rates organised in a 8 row x 8 column matrix called Q. Each rate corresponds to a transition from one health state to another shown in Figure 1 (see main paper). This matrix is called the transition rate matrix, Q. Each row (by definition) sums to zero. In general terms, each cell q(i,j) in Q represents the instantaneous rate at which a patient might “transit” from health state i to health state j at time T. There is one backward transition in this matrix, representing healing following recurrence, rate *d* in *Fig. 1*.

Given Q, we need to calculate the corresponding 8x8 transition probability matrix, P, for use in the Markov model. The cycle length of the model is one year, so a probability p(i,j) is the proportion of patients in state i at the start of a year that end up in state j at the end of the year. Each row of matrix P will sum to 1.

**Table A1** The transition rate matrix Q

|  | Not healed | Healed 1yr | Healed 2yr | Healed 3yr | Healed 4yr | Healed 5yr | Recur | Dead |
| --- | --- | --- | --- | --- | --- | --- | --- | --- |
| Not healed | -a(T)-c(T) | a(T) | 0 | 0 | 0 | 0 | 0 | c(T) |
| Healed 1yr | 0 | 0 | b(1)-c(T) | 0 | 0 | 0 | b(1) | c(T) |
| 2yr | 0 | 0 | 0 | -b(2)-c(T) | 0 | 0 | b(2) | c(T) |
| 3yr | 0 | 0 | 0 | 0 | -b(3)-c(T) | 0 | b(3) | c(T) |
| 4yr | 0 | 0 | 0 | 0 | 0 | -b(4)-c(T) | b(4) | c(T) |
| 5yr | 0 | 0 | 0 | 0 | 0 | -b(5)-c(T) | b(5) | c(T) |
| Recur | 0 | d | 0 | 0 | 0 | 0 | -d-c(T) | c(T) |
| Dead | 0 | 0 | 0 | 0 | 0 | 0 | 0 | 0 |

We begin by noting that, as 4 of the states (healed 1yr, healed 2yr, healed 3yr and healed 4yr) in *Table A1* are tunnel states, the rate matrix Q can be simplified to a 4x4 matrix, as in *Table A2*. At the end of the procedure, we will reconstitute the full 8x8 probability matrix with all the tunnel states.

*Step 1: Write down the transition matrix Q*

Dropping the (T) notation, we start with the rate matrix as a 4x4 matrix Q as in *Table A2*.

**Table A2** The transition rate matrix Q, simplified to exclude tunnel states

|  | Not healed | Healed | Recur | Dead |
| --- | --- | --- | --- | --- |
| Not healed | -a-c | a | 0 | c |
| Healed | 0 | -b-c | b | c |
| Recur | 0 | d | -d-c | c |
| Dead | 0 | 0 | 0 | 0 |

*Step 2: Derive the eigenvalues of Q*

We use a computer algebra system, Maxima[5], to calculate the eigenvalues and eigenvectors associated with this simplified matrix. The Maxima code (see Jones 2017) is:

Q: matrix([-a-c,a,0,c], [0,-b-c,b,c], [0,d,-d-c,c],[0,0,0,0]);

[evalues, evectors]: eigenvectors(Q);

This gives the eigenvalues matrix D (*Table A3*).

**Table A3** Eigenvalues matrix D

| -(b+c+d) | 0 | 0 | 0 |
| --- | --- | --- | --- |
| 0 | -(a+c) | 0 | 0 |
| 0 | 0 | -c | 0 |
| 0 | 0 | 0 | 0 |

We then use intermediate variables to represent the cells of the eigenvalues matrix. Hence the variable e represents -(b+c+d) and f represents –(a+c) (*Table A4*).

**Table A4** Representation of matrix D using intermediate variables

| e | 0 | 0 | 0 |
| --- | --- | --- | --- |
| 0 | f | 0 | 0 |
| 0 | 0 | -c | 0 |
| 0 | 0 | 0 | 0 |

As D is a diagonal matrix, the exponential of D is easy to calculate (*Table A5*).

**Table A5** Exponential of D

| Exp(e) | 0 | 0 | 0 |
| --- | --- | --- | --- |
| 0 | Exp(f) | 0 | 0 |
| 0 | 0 | Exp(-c) | 0 |
| 0 | 0 | 0 | 1 |

*Step 3. Derive the eigenvector matrix of Q*

The eigenvector matrix associated with Q (obtained from the Maxima code above) is given in *Table A6*, and we give it the name U.

**Table A6** Eigenvectors matrix U

| a | 1 | 1 | 1 |
| --- | --- | --- | --- |
| a-b-d | 0 | 1 | 1 |
| (d^2^+(b-a)d)/b | 0 | 1 | 1 |
| 0 | 0 | 0 | 1 |

We then define intermediate variables h and i to represent the cells of U (*Table A7*). Hence h = a-b-d and i = (d^2^+(b-a)d)/b

**Table A7** Representation of matrix U using intermediate variables

| a | 1 | 1 | 1 |
| --- | --- | --- | --- |
| h | 0 | 1 | 1 |
| i | 0 | 1 | 1 |
| 0 | 0 | 0 | 1 |

*Step 4. Calculate the inverse of U*

The inverse of matrix U (*Table A8*) can be easily calculated by using the “invert” command in Maxima[5].

**Table A8** Inverse of U = U^-1^

| 0 | -1 | 1 | 0 |
| --- | --- | --- | --- |
| i-h | a-i | h-a | 0 |
| 0 | i | -h | h-i |
| 0 | 0 | 0 | 1 |

*Step 5. Calculate the transition probability matrix P = U.exp(D).U^-1^*

The probability transition matrix P is then straightforward to calculate in Maxima[5] as the matrix product of the three matrices: U, exp(D) and the inverse of U as calculated previously (*Table A9*).

**Table A9** Probability matrix P

|  | Not healed | Healed | Recur | Dead |
| --- | --- | --- | --- | --- |
| Not healed | Exp(f) | [Exp(-c)i+Exp(f)(a-i)-Exp(e)a]/(i-h) | [Exp(f)(h-a)-Exp(-c)h+Exp(e)a]/(i-h) | 1-Exp(-c) |
| Healed | 0 | [Exp(-c)i-Exp(e)h]/(i-h) | [Exp(e)h-Exp(-c)h]/(i-h) | 1-Exp(-c) |
| Recur | 0 | [Exp(-c)i-Exp(e)i]/(i-h) | [Exp(e)i-Exp(-c)h]/(i-h) | 1-Exp(-c) |
| Dead | 0 | 0 | 0 | 1 |

*Table A9* can be re-written by defining intermediate variables, so that P(1,1) represents exp(f) and so on, see *Table A10*.

**Table A10** Probability matrix P, expressed in terms of intermediate variables

|  | Not healed | Healed | Recur | Dead |
| --- | --- | --- | --- | --- |
| Not healed | P(1,1) | P(1,2) | P(1,3) | P(4) |
| Healed | 0 | P(2,2) | P(2,3) | P(4) |
| Recur | 0 | P(3,2) | P(3,3) | P(4) |
| Dead | 0 | 0 | 0 | 1 |

Finally, we need to reconstitute the 8x8 matrix with the tunnel states following healing. The rates of recurrence in each tunnel state at time t since the ulcer healed are calculated using the Weibull formula. We note the definition of a tunnel state. In a tunnel state with a healed ulcer, during the next year the patient will either die, the ulcer will recur, or the patient will pass to the next time-period state (the ulcer remains healed and the time elapsed since healing advances by one year). Thus given a patient in state 2 (healed ulcer) at year t following healing, then during the following year the ulcer can recur with probability P(2,3)_t_ , the patient can die with probability P(4), or the patient will pass to the next tunnel state (corresponding to time t+1 following healing) with probability P(2,2)_t_ (*Table A11*). These probabilities can all be calculated using the formulas set out in the previous *Tables A1–A9*.

All the transition probabilities in *Table A11*, expressed algebraically in terms of their constituent intermediate variables as set out in the previous *Tables A1–A9*, are used in the Markov model spreadsheet. The model spreadsheet is made available for researchers. http://dx.doi.org/10.17632/7634sv27zp.1

**Table A11** Reconstituted 8x8 probability matrix with tunnel states

|  | Not healed | Healed 1yr | Healed 2yr | Healed 3yr | Healed 4yr | Healed 5yr | Recur | Dead |
| --- | --- | --- | --- | --- | --- | --- | --- | --- |
| Not healed | P(1,1) | P(1,2) | 0 | 0 | 0 | 0 | P(1,3) | P(4) |
| Healed 1yr | 0 | 0 | P(2,2)_1_ | 0 | 0 | 0 | P(2,3)_1_ | P(4) |
| 2yr | 0 | 0 | 0 | P(2,2)_2_ | 0 | 0 | P(2,3)_2_ | P(4) |
| 3yr | 0 | 0 | 0 | 0 | P(2,2)_3_ | 0 | P(2,3)_3_ | P(4) |
| 4yr | 0 | 0 | 0 | 0 | 0 | P(2,2)_4_ | P(2,3)_4_ | P(4) |
| 5yr | 0 | 0 | 0 | 0 | 0 | P(2,2)_5_ | P(2,2)_5_ | P(4) |
| Recur | 0 | P(3,2) | 0 | 0 | 0 | 0 | P(3,3) | P(4) |
| Dead | 0 | 0 | 0 | 0 | 0 | 0 | 0 | 1 |

**References for Appendices**

1. Siebert U, Alagoz O, Bayoumi AM, Jahn B, Owens DK, Cohen DJ, et al. State-Transition Modeling: A Report of the ISPOR-SMDM Modeling Good Research Practices Task Force-3 Background to the Task Force. Value Heal [Internet]. 2012;15:812–20. Available from: http://dx.doi.org/10.1016/j.jval.2012.06.014

2. Jones E, Epstein D, García-Mochón L. A Procedure for Deriving Formulas to Convert Transition Rates to Probabilities for Multi-State Markov Models. Med Decis Mak [Internet]. 2017; Available from: http://journals.sagepub.com/doi/10.1177/0272989X17696997

3. Mauck KF, Asi N, Undavalli C, Elraiyah TA. Systematic review and meta-analysis of surgical interventions versus conservative therapy for venous ulcers. J Vasc Surg [Internet]. Society for Vascular Surgery; 2013;60(2):60S–70S.e2. Available from: http://dx.doi.org/10.1016/j.jvs.2014.04.059

4. Briggs A, Claxton K, Sculpher M. Decision modelling for health economic evaluation. Oxford: OUP; 2006. 1-237 p.

5. Maxima, a Computer Algebra System. [Internet]. 2016. Available from: http://maxima.sourceforge.net/

**Fig. S1** Incremental cost-effectiveness ratio (ICER; cost per quality-adjusted life-year gained) over time. Difference in cost: difference in overall cost in euros per patient between surgery and compression therapy only. Dominates: the overall costs of surgery are lower and the health gain is greater than compression therapy alone

**Table S1** Results of analysis of co-variance

| Parameter | Base case value | Contribution to the overall uncertainty in the difference in cost between surgery and compression therapy only | Contribution to the overall uncertainty in the difference in cost between surgery and compression therapy only |
| --- | --- | --- | --- |
| Relative risk of recurrence after surgery versus compression | 0.67 (0.41-1.10) | 86% | 87% |
| Relative risk of healing after surgery versus compression | 1.04 (0.98-1.09) | 6% | 6% |
| EQ-5D associated with unhealed ulcer | 0.64 (0.60-0.68) | 3% | 3% |
| Others (jointly) |  | 5% | 4% |
